# Comparative trends in oral cancer burden across the globe, China, Europe, the US, Southeast Asia, and Africa (1990–2021): a GBD 2021 analysis

**DOI:** 10.1186/s41182-025-00882-7

**Published:** 2025-12-21

**Authors:** Songling Hu, Bin Yang, Tian Yu, Can Wang, Ling Huang, Xiaofei Li, Liya Jiang, Qingling Hu, Jing Zhu

**Affiliations:** 1 Department of Preventive Dentistry, Shanghai Stomatological Hospital, School of Stomatology, Fudan University, Shanghai, 200001 China; 2 Shanghai Key Laboratory of Craniomaxillofacial Development and Diseases, Fudan University, Shanghai, 200001 China; 3 Department of Stomatology, Shandong University Qilu Hospital Dezhou Hospital (Dezhou People’s Hospital), Dezhou, Shandong, 253000 China

**Keywords:** Oral cancer, GBD 2021, Risk factors, Regional comparison

## Abstract

**Background:**

Oral cancer including lip, oral cavity cancer contributes to cancer burden importantly in the world. It is crucial for effective policy planning to comprehensively evaluate oral cancer burden regionally.

**Methods:**

The incidence, mortality, and disability-adjusted life years (DALYs) due to oral cancer from 1990 to 2021 were estimated according to Global Burden of Disease (GBD) 2021 methods. The GBD comparative risk assessment framework was used to estimate the proportion of deaths and DALYs for oral cancer attributable to smoking, tobacco, and alcohol consumption in 2021.

**Results:**

The male-to-female ratio of age-standardized incidence rate (ASIR) for oral cancer was 2.99 in China, 2.7 in Europe, 2.24 in the United States, 1.73 in Southeast Asia, and 1.51 in Africa. The corresponding ratios of age-standardized mortality rate (ASMR) for oral cancer were 3.82, 3.16, 2.45, 1.89, and 1.60 respectively. Key risk factors for oral cancer-related deaths and DALYs varied by region and showed distinct age- and sex-stratified patterns. In China, tobacco was the primary contributor, accounting for 51.4% of oral cancer deaths in men, with a higher impact among older males aged ≥ 55 years. In Europe and the United States (US), alcohol consumption dominated, contributing a larger proportion of deaths in younger men (20–54 years) and showing higher attributable fractions than smoking in these age groups. In Southeast Asia, chewing tobacco was the major driver, responsible for 48.79% of oral cancer deaths in women, with this proportion exceeding 50% in females aged ≥ 55 years. Among men in Southeast Asia, smoking was the predominant risk factor for oral cancer mortality.

**Conclusions:**

The burden of oral cancer exhibits distinct temporal and regional variations, with significant differences in incidence, mortality, and DALYs across global regions. Such differences are strongly associated with region-specific risk factor patterns, and these patterns also vary by age and sex. These insights highlight the need for targeted prevention strategies tailored to regional, age, and sex characteristics, including anti-smoking interventions in older Chinese men, alcohol control measures in younger European and American men, and efforts to reduce chewing tobacco use among older Southeast Asian women, to effectively mitigate the global burden of oral cancer.

## Background

Oral cancer is defined as any malignant neoplasm of the lip and oral cavity corresponding to the International Classification of Diseases [ICD-10] codes C00-C081 [1]. In 2022, oral cancer was the sixteenth most common cancer globally, the fifteenth most common cause of cancer deaths, while in low and middle-income countries, oral cancer was the fifth most common cancer, and the 8th most common cause of cancer deaths [2]. In 2021 about 9.9 million people died due to cancer worldwide according to Global Burden of Disease Study 2021 and 2.1% of global cancer deaths were caused by lip and oral cavity. The survival of lip, oral cavity cancer varies broadly around the world and survivors may generally have substantial reductions in their quality of life [3, 4]. As shared risk factors with other chronic noncommunicable diseases, oral cancer has high mortality and is distributes around the world unevenly with more than 50% in low-income countries due to multifactorial differences in access to early-stage detection and effective therapies along with potential differences in risk factor exposure patterns [4]. Some established oral cancer risk factors such as tobacco, alcohol, and betel quid consumption were demonstrated in previous reports to increase oral cancer risk dose- and time-dependently to contribute to the incidence, mortality, disability-adjusted life years (DALYs) burden of oral cancer [4, 5]. Especially relevant in certain geographic areas of the world, human papillomavirus (HPV) infection is known as one of established risk factors for oral cavity cancer [5–7]. HBV infection [8], poor oral hygiene and carbohydrate-rich foods can also act as high risk factors to increase oral cancer development [9].

The World Health Organization (WHO) adopted an oral health resolution at the 2021 World Health Assembly, which explicitly calls for advancing the prevention and control of oral cancer by comprehensively assessing and comparing its disease burden associated with various modifiable risk factors [4, 10]. This resolution provides action-oriented guidance for global and national efforts to address oral cancer. With the rapid socioeconomic development, the characteristics of China’s cancer spectrum are showing a tendency towards those of developed countries’ cancer spectra [11], and the same is true for oral cancer. It is noteworthy that the characteristics of oral cancer incidence trends in China may differ from those in regions such as Europe, North America, Southeast Asia, and Africa. Understanding the risk factors of oral cancer across different regions serves as a critical prerequisite for formulating effective prevention and control strategies. Conducting a detailed analysis and comparison of data including oral cancer incidence, mortality, DALYs and relevant risk factors across the aforementioned regions can provide a scientific and valuable reference for China’s oral cancer prevention and control efforts.

The Global Burden of Diseases (GBD) study encompasses diseases, injuries, and risk factors, and is integrated into global disease estimation frameworks to provide a reference for assessing the effectiveness of disease control programs, establishing benchmarks for progress, and monitoring the magnitude of disease burden as well as its demographic characteristics and spatiotemporal variations [4]. The aim of this study was to extract and systematically analyze the incidence, mortality, DALYs, and risk factors for lip and oral cavity cancer from 1990 to 2021 across global, Chinese, European, the United States (US), Southeast Asian, and African populations. A comprehensive comparative assessment was conducted by benchmarking oral cancer against all cancers combined in terms of numbers of cases, age-standardized incidence rates (ASIR), and age-standardized mortality rates (ASMR), to identify its unique epidemiological trends and geographic disparities. Additionally, we assessed the oral cancer burden attributable to tobacco and alcohol use based on GBD 2021 estimates.

## Methods

### Data sources

Data on the incidence, mortality, and DALYs for lip and oral cavity cancer were extracted from the Global Burden of Disease Study 2021 (GBD 2021) via the Institute for Health Metrics and Evaluation (IHME) online results tool [12]. It is important to note that the IHME results tool, while currently updated to the GBD 2023 version, continues to provide complete access to the historical GBD 2021 data used in this study. Cause of death, mortality, DALYs, years of life lost and years of life lived with disability by age and sex for 87 risk factors and risk factor combinations for 7 super-regions, 21 world regions (21 total), and 204 countries or territories as estimated annually by the GBD project [13, 14], and form the basis of our analysis. In this study, we presented the 2021 estimates of DALYs per 100,000 population for oral cancer, stratified by sex and age, for China, Europe, the US, Southeast Asia, and Africa. Furthermore, to reflect trends in the oral cancer burden, we calculated the percentage changes and ranking shifts in both all-age and age-standardized DALYs from 1990 to 2021, using the GBD reference population.

### Estimating disease burden and attributable risk factors

The estimates reported for adults aged 20 years and older are presented by sex, 5-year age groups (20–24, 25–29, 30–34, … ≥ 95 years), in China, and by region for the years 1990 to 2021 according to the method used in a previous study [15]. The GBD study models mortality-to-incidence ratios (MIRs) using cancer registry data; these MIRs are then used to estimate incidence by dividing the final modelled mortality estimates by these corresponding MIRs [16]. The Comparative Risk Assessment (CRA) framework from the GBD 2021 study was used to estimate the proportion of deaths and DALYs in 2021 attributable to the selected risk factors [17]. Furthermore, the GBD 2021 study conducted a comprehensive assessment of the disease burden attributable to various risk factors and their combinations at global, regional, and national scales. Based on the risk-outcome pairs that were assessed by the GBD study as meeting the World Cancer Research Fund (WCRF) grades of “convincing evidence” or “probable evidence” and that were derived from meta-analyses, case–control studies, and prospective cohort studies, we selected smoking, alcohol consumption, and other relevant factors [13, 14]. The theoretical minimum risk exposure level for smoking and chewing tobacco was that all individuals were lifelong nonusers and for alcohol use was an estimated daily consumption of 0 to 10 g [18].

### Statistical analysis

Numbers, percents and rate data were obtained from GBD. The data were analyzed using GraphPad Prism 8. To indicate the direction and magnitude of the trends, the annual percent change was calculated in this study. The term “decrease” or “increase” was used when the slope of the trends was significantly different from zero. *P* value less than 0.05 was considered statistically significant.

## Results

### Incidence and mortality of oral and all cancers in global and regional contexts

**Fig. 1:**
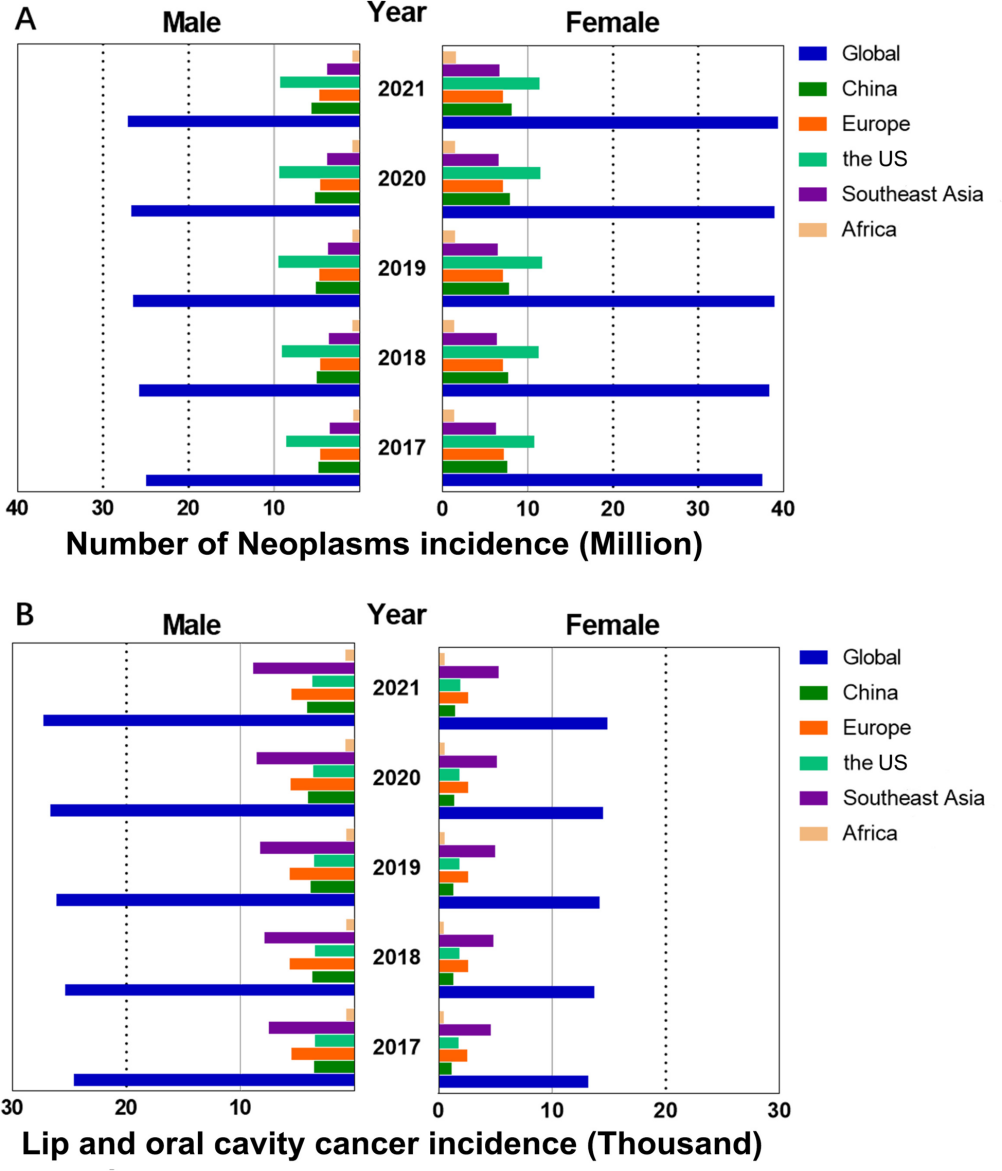
Number of new cancer (**A**) and oral cancer cases (**B**) globally, in China, Europe, the US, Southeast Asia and Africa in 2021

Based on the estimates from the GBD 2021 study, 66,479,607 new cancer cases were diagnosed in the world in 2021. Among these, as many as 421,577 cases were oral cancers, with incidence of 5.34 per 100,000. In China, 13,664,748 cancer cases occurred and 56,359 cases including 41,648 males and 14,710 females. The number of males suffering from oral cancer is about 1.84 times that of women in the world, and 2.83 times that of females in China (Fig. 1).

**Fig. 2:**
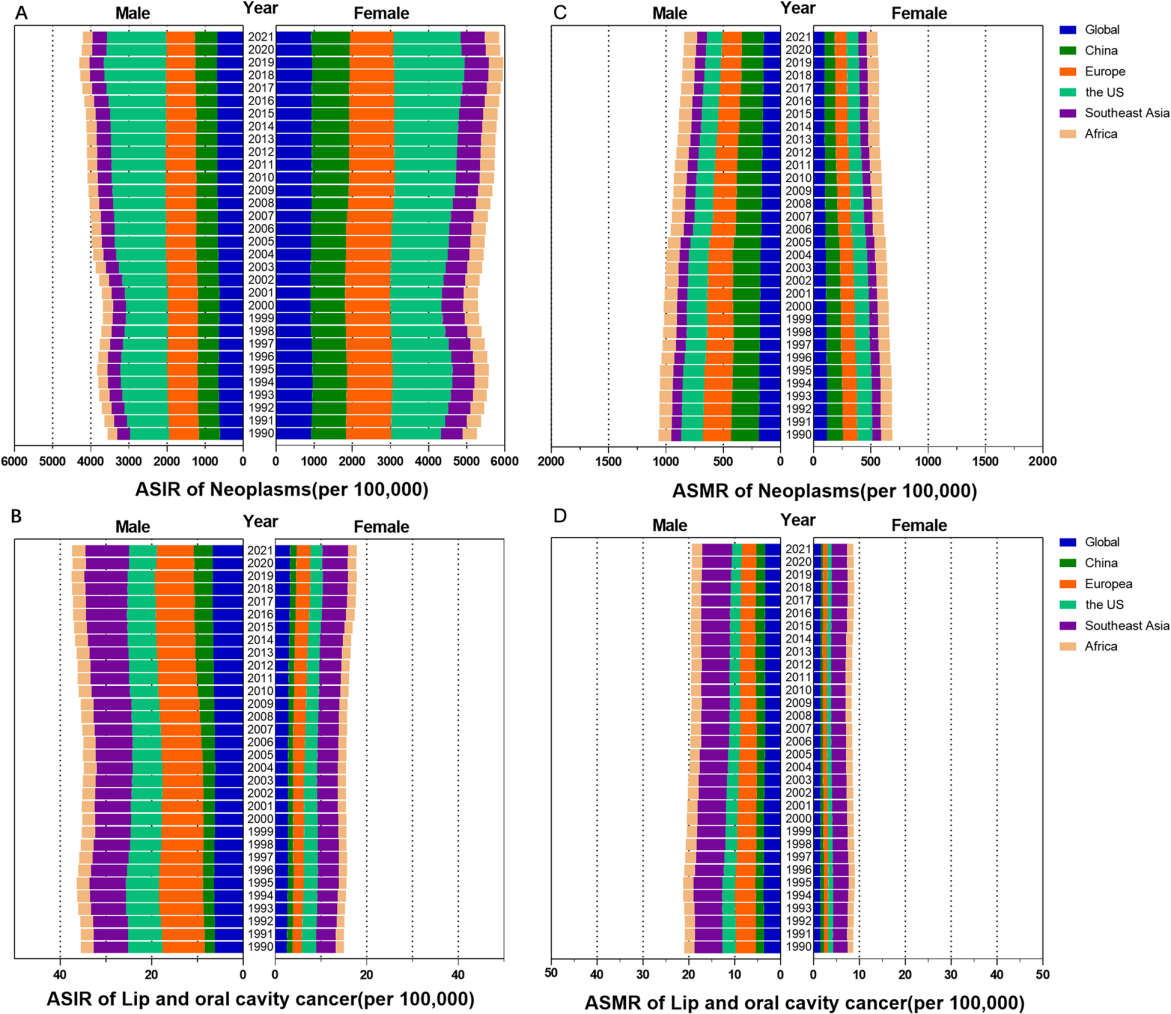
ASMR and ASIR in males, females, and both sexes from 1990 to 2021. **A** ASMR was analyzed globally, in China, in Europe, in the US, in Southeast Asia and in Africa. **B** ASMR of lip and oral cavity cancer. **C** ASIR was analyzed globally, in China, in Europe, in the US, in Southeast Asia and in Africa. **D** ASIR of lip and oral cavity cancer

The ASIR for all cancers was 790.33 per 100,000 population. Women showed a higher ASIR than men (923.44 per 100,000 population for women vs. 673.09 per 100,000 population for men) worldwide. In both sexes combined, oral cancer showed the ASIR as 4.88 per 100,000 population, and the ASIR for men was 6.65 per 100,000 population and the ASIR for women was 3.28 per 100,000 population. In China, the ASIR for all cancers was 790.17 per 100,000 population and women showed a higher ASIR than men (1,014.80 per 100,000 population for women vs. 599.70 per 100,000 population for men). For oral cancer, the ASIR was 2.68 per 100,000 population in both sexes, and men showed a higher ASIR of 4.13 per 100,000 population compared with women, who had an ASIR of 1.38 per 100,000 population (Fig. 2A, B).

The oral cancer ASIR demonstrated considerable regional variation, with the highest burden in Southeast Asia (7.55) and the lowest in Africa (2.41). Across all regions, the incidence was consistently higher in men, with specific rates for men versus women being 8.13 vs. 3.01 in Europe, 5.97 vs. 2.66 in the US, 9.64 vs. 5.57 in Southeast Asia, and 2.92 vs. 1.94 in Africa.

Among all the regions including China, the US showed the highest, Europe ranked second and Africa showed the lowest all cancer ASIR. As for oral cancer ASIR in both sexes, Southeast Asia region showed the highest, Europe ranked second and Africa showed the lowest similarly. China showed the second ASIR of oral cancer. The ratio of oral cancer ASIR in men to women was the highest in China (2.99), Europe with 2.7 ranks second, 2.24 in the US, Africa showed the lowest as 1.51 and Southeast Asia region showed the second lowest as 1.73 which suggests that oral cancer is more common among both sexes in Southeast Asia, where the oral cancer ASIR is the highest.

**Fig. 3:**
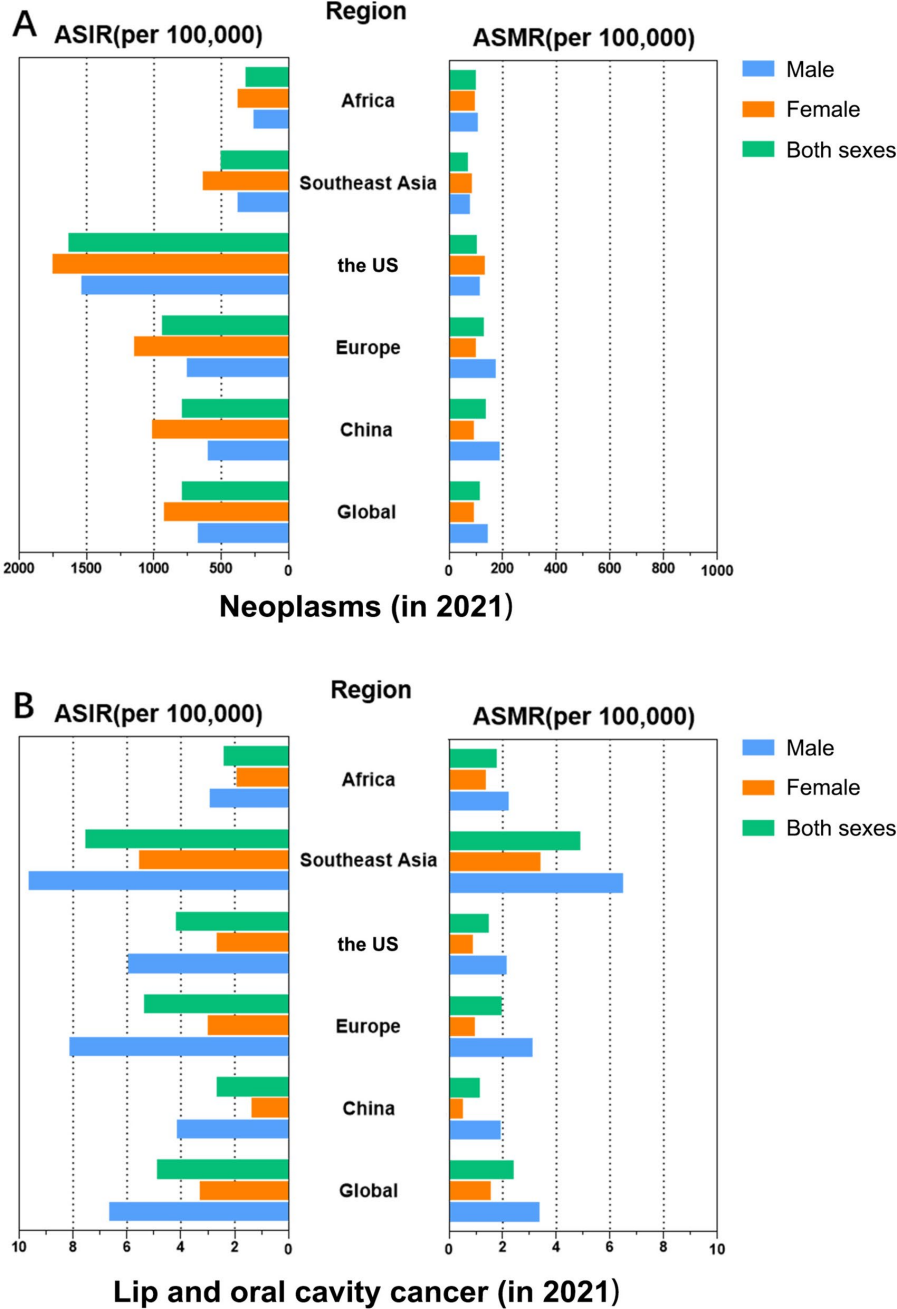
ASMR, in males, females and both sexes of all cancer in 2021. **A** ASMR was analyzed globally, in China, in Europe, in the US, in Southeast Asia and in Africa. **B** ASMR for lip and oral cavity cancer

The number of all cancer-related deaths was 9,888,413, and the number of oral cancer-related deaths was 208,379 worldwide in 2021, based on the GBD study. The global ASMR for all cancers was 116.49 per 100,000 population. In contrast, the ASMR for oral cancer was 2.42 per 100,000 population, with a male-to-female ratio of 3.39 (males) versus 1.56 (females) (Fig. 2C, D). A marked geographical variation was observed in the oral cancer ASMR. Southeast Asia bore the highest burden at 4.90 per 100,000, followed by Africa (1.77), Europe (1.95), the United States (1.47), and China, which had the lowest rate (1.15) (Fig. 3). This pattern persisted when analyzed by sex, albeit with notable variations in magnitude. In men, the ASMR decreased from Southeast Asia (6.46) to Europe (3.10), Africa (2.21), the United States (2.16), and China (1.91). A parallel trend was observed in women, with the highest rate in Southeast Asia (3.41), followed by Africa (1.38), Europe (0.98), the United States (0.88), and the lowest in China (0.50). Paradoxically, while China bore the highest ASMR for all cancers combined, it recorded the lowest for oral cancer; this low incidence was accompanied by the largest sex disparity in mortality, with a male-to-female ratio of 3.82, followed by Europe (3.16), the US (2.45), Southeast Asia (1.89), and Africa (1.60).

### The burden of oral cancer over time in China, Europe, the US, Southeast Asia and Africa

**Fig. 4:**
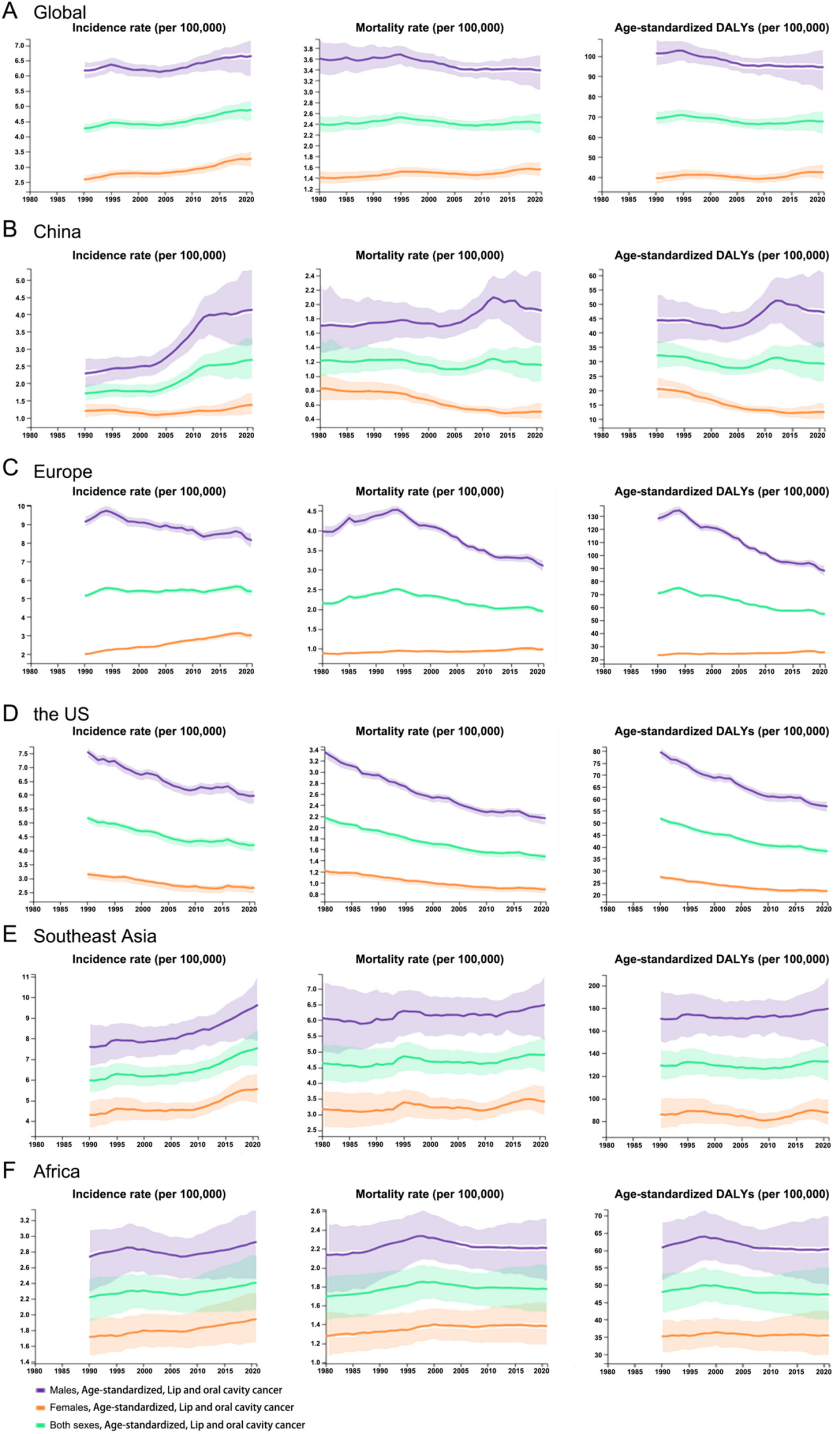
The ASMR, age-standardized DALYs and ASIR in males, females and both sexes for oral cancer. **A** Global, **B** China, **C** Europe, **D** the US, **E** Southeast Asia, **F** Africa

From 1990 to 2021, an increasing trend in ASIR, ASMR and age-standardized DALYs rates for oral cancer per 100,000 population in women and in both sexes worldwide was showed (Fig. 4A). As for men, the ASIR increased while the ASMR and age-standardized DALYs rates decreased globally. In China between 1990 and 2021, the overall (both sexes) ASIR for oral cancer increased significantly, while the overall ASMR and age-standardized DALYs remained stable (Fig. 4B). This overall stability in mortality and burden masked divergent sex-specific trends: all three measures (ASIR, ASMR, and age-standardized DALYs) increased in males, whereas in females, the ASIR increased while the ASMR and age-standardized DALYs rates decreased. In the region of Europe, the ASIR increased in both sexes while the ASMR and age-standardized DALYs rates decreased due to oral cancer. All the ASIR, ASMR and age-standardized DALYs rates tended to increase in women while decreasing in men due to oral cancer from 1990 to 2021 over time (Fig. 4C). In the region of the US, all the ASIR, ASMR and age-standardized DALYs rates per 100,000 population in women, men as well as in both sexes were decreased due to oral cancer from 1990 to 2021 over time (Fig. 4D). In Southeast Asia, all the ASIR, ASMR and age-standardized DALYs rates in women, men as well as in both sexes were decreased due to oral cancer from 1990 to 2021 over time (Fig. 4E). The ASIR increased in men, women and both sexes correspondingly while the ASMR and age-standardized DALYs rates per 100,000 population did not show significantly variation in men, women and both sexes from 1990 to 2021 due to oral cancer in Africa over time (Fig. 4F).

**Fig. 5:**
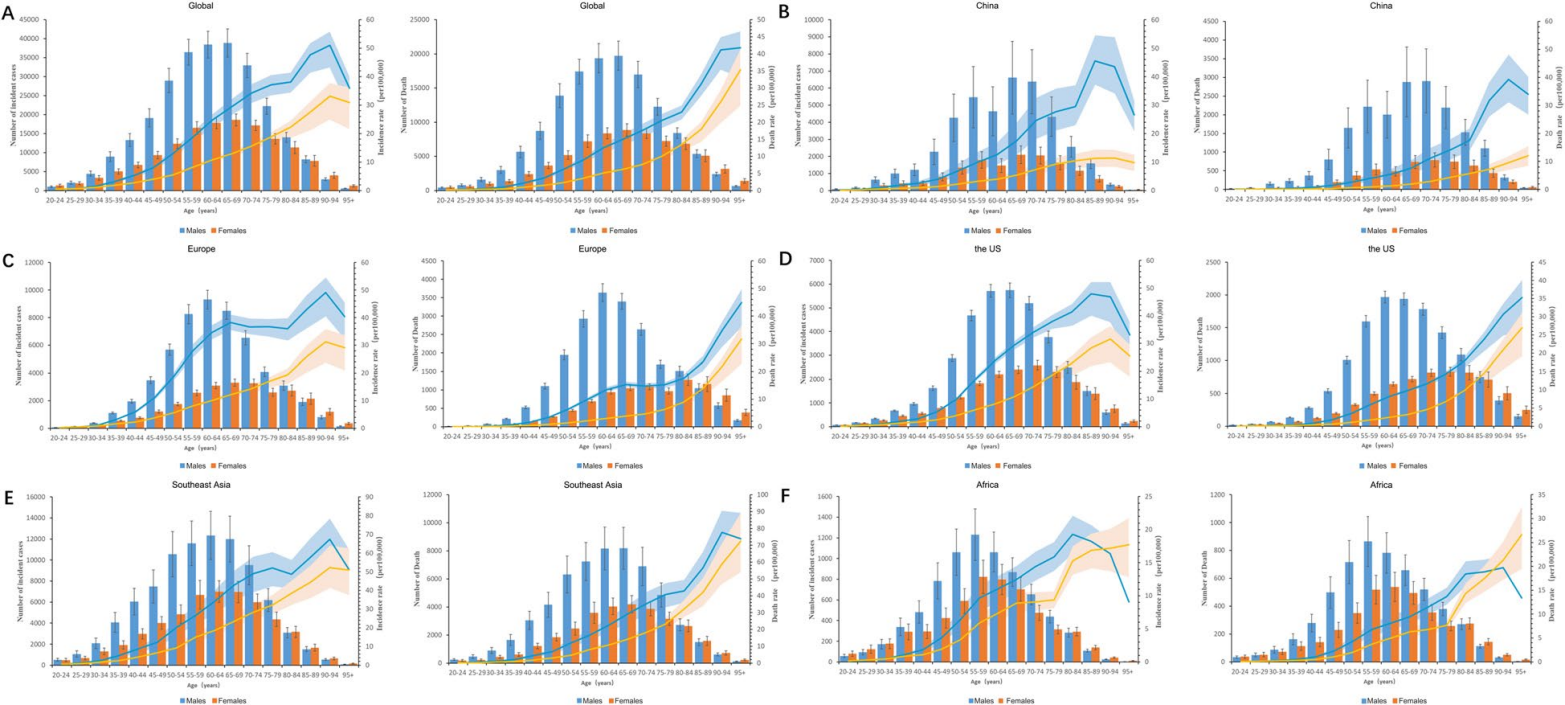
Absolute cases and deaths, and age-specific incidence and mortality rates for oral cancer by age group and sex in 2021. **A** Global, **B** China, **C** Europe, **D** the US, **E** Southeast Asia, **F** Africa

Figure 5 illustrates the 2021 burden of oral cancer through absolute cases, deaths, and age-specific rates. The distributions are detailed by age group and sex at the global level and for key regions (China, Europe, the US, Southeast Asia, and Africa). The oral cancer age-specific rates for incidence, mortality, and DALYs were higher among women than among men in 20 to 24, 90 to 94 and persons more than 95 years old while in other age groups, the oral cancer age-specific rates for incidence, mortality, and DALYs were higher among men than among women. In China, the oral cancer age-specific rates for incidence, mortality, and DALYs were higher among women than among men only in persons more than 95 years old and in the other age groups, the oral cancer age-specific rates for incidence, mortality, and DALYs were higher among men than among women. The oral cancer age-specific rates for incidence, mortality, and DALYs were higher among women than among men in more than 85 years old in the region of Europe, in persons more than 90 years old in the US, in persons more than 85 years old Southeast Asia and in 20 to 29, and persons more than 80 years old in Africa. In the other age groups, the oral cancer age-specific rates for incidence, mortality, and DALYs were higher among men than among women in Europe, the US, Southeast Asia and Africa.

### Global trends in oral cancer burden, changes in China, and comparison with Europe, the US, Southeast Asia and Africa

**Fig. 6:**
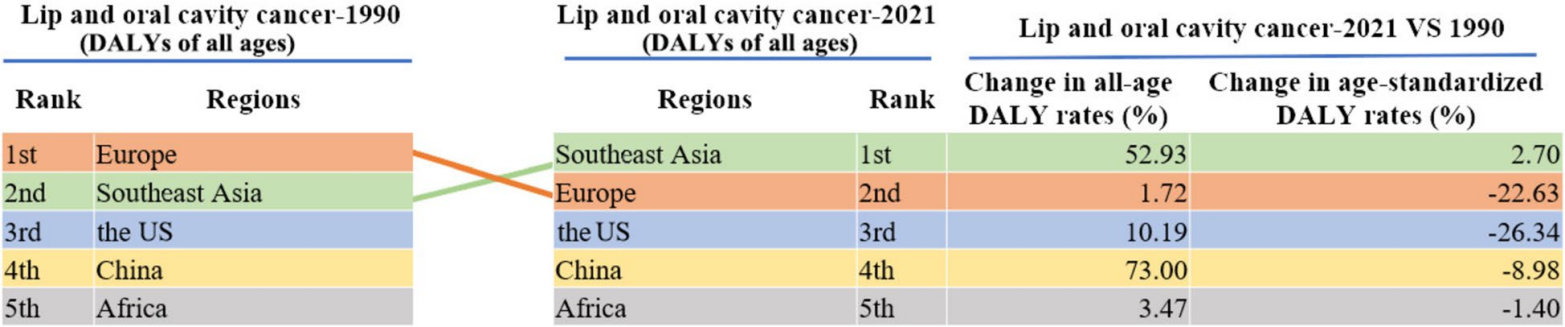
Regional trends in all-age DALYs rates attributable to oral cancer, 1990–2021

Between 1990 and 2021, the age-standardized DALYs rate in the world decreased by 2.3%, from 69.27 (95% uncertainty interval [UI] = 72.96–65.92) per 100,000 population in 1990 to 67.71 (95% UI = 73.17–61.32) per 100,000 population in 2021 due to oral cancers. The rate decreased by 6.8% (from 101.41 to 94.55) in males and increased by 7.6% (from 39.59 to 42.58) in females due to oral cancer worldwide. For the disease burden attributable to oral cancers, the age-standardized DALYs rate in China decreased by 9.6%, from 32.09 (95% UI = 37.01–27.04) per 100,000 population in 1990 to 29.02 (95% UI = 36.49–23.18) per 100,000 in 2021. The ranking changes in China and comparison with Europe, the US, Southeast Asia and Africa in all‐age DALYs rates attributable to oral cancer are summarized in Fig. 6. The age‐standardized DALYs rate increased by 2.7% in the region of Southeast Asia, along with a decrease of 26.3% in the US, 22.6% in Europe and 1.39% in Africa. Among these regions, China ranks the second-lowest for decreasing the age‐standardized DALYs rate (by 9.6%) from 1990 to 2021 due to oral cancers.

### Risk factors: population attributable fraction

**Fig. 7:**
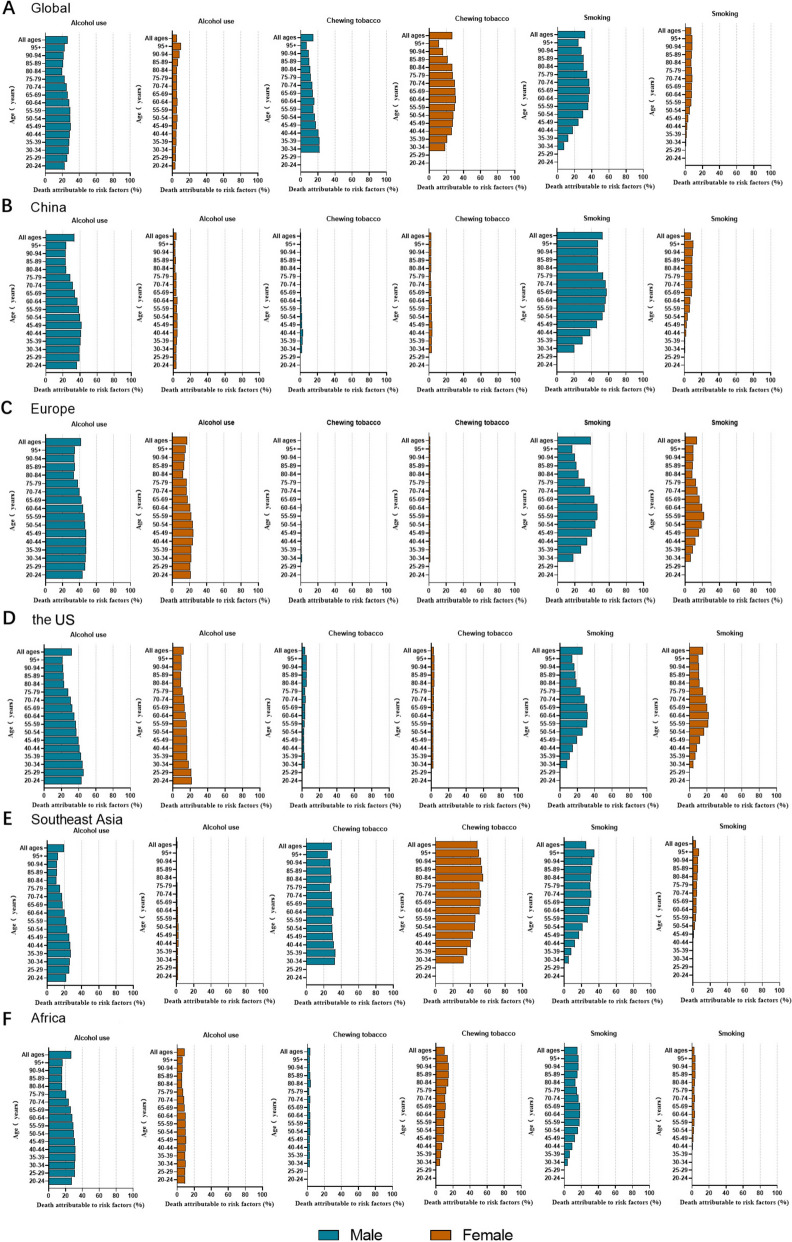
Proportion of oral cancer deaths attributable to risk factors in 2021, by gender. **A** Global, **B** China, **C** Europe, **D** the US, **E** Southeast Asia, **F** Africa

Figure 7 shows the proportion of oral cancer deaths attributable to alcohol and tobacco consumption in 2021. Among males, tobacco smoking and alcohol consumption were responsible for a large proportion of oral cancer deaths globally (31.9% [95% UI, 22.7%-40.5%] and 25.9% [95% UI, 20.3%-30.5%], respectively). For males in the younger age group (20 to 54 years old), alcohol consumption was a more important risk factor than smoking and in the older age group (≥ 50 years old) smoking was a more important risk factor than alcohol consumption. Among females, the proportion of risk-attributable oral cancer deaths globally showed no significant difference between smoking and alcohol consumption. It was reported that the highest proportion of risk-attributable oral cancer deaths globally were due to chewing tobacco among women [4]. In China, for males, tobacco smoking and alcohol consumption were responsible for 51.4% and 32.0% oral cancer deaths and in the younger age group alcohol consumption was more important, while in the older age group, alcohol was a less important risk factor than smoking. Smoking was a more important risk factor for oral cancer deaths in the Southeast Asian region than alcohol consumption. In Europe, the US and Africa, alcohol consumption was more important risk factor than smoking especially in the younger age groups attributed to oral cancer deaths in 2021. It is worth noting that smoking was more important risk factor attributed to oral cancer deaths in the older age group and among males in China and Southeast Asia as well as chewing tobacco among females.

## Discussion

The oral health agenda made a considerable step forward with the adoption of a resolution on oral health: the WHA74.5 resolution adopted by WHO at the World Health Assembly in 2021. This resolution led to the development of a global strategic action plan for oral health, which was adopted by member states in 2022 and sets the strategic direction for achieving universal oral health coverage for all by 2030 [19]. The specific interventions for oral cancer involving screening in high-risk groups to link cancer treatment to timely comprehensive diagnosis were advocated based on a global noncommunicable diseases action plan for 2013 to 2030 by the WHO [4]. It provided an opportunity to improve oral cancer patient outcomes collectively and globally through many efforts [20, 21].

In the present study, the burden of oral cancer, that is lip and oral cavity cancer was updated and comprehensively overviewed globally and in China and comparison with Europe, the US, Southeast Asia and Africa, based on the GBD 2021 estimates from 1990 to 2021. Specifically, this study profiles the burden attributable to its risk factors through an analysis of deaths, ASIRs, ASMRs, and DALYs, stratified by sex, age, and time period. The findings are intended to inform intervention strategies globally and in key WHO regions, including China, Europe, the US, Southeast Asia, and Africa. Disparities in ASIRs and ASMRs and trends across the time, age groups and regions suggested that risk factors such as smoking, chewing tobacco and alcohol consumption were primary underlying causes of oral cancer mortality. In high-sociodemographic index (SDI) regions (such as Europe and the US), although the incidence rate of oral cancer may remain at a relatively high level due to the popularization of early screening and improved health awareness, the early diagnosis and standardized treatment supported by a sound medical system, often keep the mortality rate and DALYs at a relatively low level. Their risk factors are more concentrated in specific lifestyles, such as alcohol abuse among young men, which is closely related to the sociocultural environment and consumption patterns in high-SDI regions. In contrast, low-SDI regions (such as parts of Africa and Southeast Asia) face a more severe burden of oral cancer. The lack of medical resources leads to delayed diagnosis and treatment gaps, resulting in persistently high mortality rates and DALYs. The risk factors show diverse characteristics. In addition to smoking, traditional habits such as tobacco chewing are more common among women, and there is a lack of effective tobacco control and health education interventions. Notably, an upward trend in the ASIR was observed in China and Southeast Asia, underscoring a growing disease burden in these populous regions. Furthermore, improvements in diagnostic capabilities and cancer registry systems in these regions likely enhance case detection, contributing to the observed rise in recorded incidence. Smoking, chewing tobacco and alcohol consumption may be crucial targets to decrease the burden of oral cancer in the future as their substantial roles in contributing to oral cancer deaths and DALYs in the world.

Based on the GBD 2021 estimates, ASIR, ASMR and age-standardized DALYs rates for oral cancer were higher among males than females in China as well as Europe, the US, Southeast Asia and Africa. Although ASIR, ASMR and age-standardized DALY rates for oral cancer in China were not higher than other regions included in the present study, it's particularly obvious that the ratio of ASIR for oral cancer in males to that in females was the highest for China and was followed by Europe, the US, Southeast Asia and Africa in that order. Furthermore, Southeast Asia had the highest ASIR, ASMR, and age-standardized DALY rates for oral cancer among the included regions. The estimates indicate that tobacco (both smoking and chewing) and alcohol use were the leading risk factors for global oral cancer deaths and DALYs, a finding consistent with previous studies. In addition to these overarching global risks, our analysis further highlighted several distinct high-risk patterns. Notably, smoking was a major contributor among males aged 55 and older in China; similarly, alcohol consumption significantly impacted males aged 54 and younger in Europe, the US, and Africa, while chewing tobacco was a leading factor among females aged 55 and older in Southeast Asia [4]. In general, the risk factors mainly including tobacco and alcohol were highlighted by the present study and all the results suggested that the significance of risk factor such as smoking, chewing tobacco and alcohol may be correlated with sex, age and regional distribution.

Our study confirms that tobacco smoking is a significant risk factor for oral cancer. Although the WHO Framework Convention on Tobacco Control (FCTC) (2003) has driven significant progress in tobacco control over the past decades, the variable global implementation of its measures means the tobacco epidemic is far from over [21, 22]. Our findings, which show that smoking remained a leading cause of oral cancer deaths and DALYs in 2021, provide critical evidence reinforcing the necessity of fully implementing the FCTC. Furthermore, other forms of tobacco use, such as chewing tobacco, contribute significantly to the oral cancer burden, especially among females in Southeast Asia. The GBD 2021 estimates revealed that chewing tobacco was responsible for 36.41% (95% UI, 28.80%-43.95%) of global oral cancer deaths in both sexes. Among females, the attributable fraction was substantially greater, at 48.79% (95% UI, 38.97%-58.40%). Notably, in females aged ≥ 55 years, this figure surpassed 50% (95% UI, 40.77%-61.24%) in 2021. DALYs (Disability-Age standardized) due to oral cancer among females in the older age group (≥ 55 years old) at 50.12% were also more than in the younger age group (from 20 to 54 years old) at 37.8%. It was different from smoking tobacco gradually decreasing presently, chewing tobacco as the prevalence of risk factor critically contribute to oral cancer burden in females has been constant in recent decades [23]. All the results from present and previous studies suggested that initiatives of tobacco control, including smoking and chewing tobacco, need to be expanded regionally, which may be the local challenges and opportunities to conduct initiatives in world regions where chewing tobacco habits are common.

It was indicated by the results in the present study that alcohol contributed to a large proportion of oral cancer deaths and DALYs in 2021, particularly in younger males. Our results indicated that for males aged 20–54, alcohol was a more significant risk factor for oral cancer than smoking in Europe and the US, whereas the opposite was true in China, Southeast Asia, and Africa. This geographical variation in the leading risk factor is consistent with previous studies linking alcohol's attributable risk to the SDI spectrum [24]. It was also suggested in our present study that alcohol accompanied with smoking tends to have independent besides synergistic effects [25] on the risk of oral cancer developing. Furthermore, we observed that the uncertainty intervals for estimates of ASMR, age-standardized DALYs, and ASIR were generally narrower in Europe and the US compared with other regions. This pattern may be explained by the more mature cancer surveillance systems in these high-SDI regions, where data sources are more comprehensive and classification is more detailed. These advantages provide higher-quality input data for GBD modeling, which reduces statistical uncertainty and consequently yields more precise estimates.

It was also suggested that inequities exist in the oral cancer burden globally, including in all developed, developing and underdeveloped countries or regions, such as China, Europe, the US, Southeast Asia and Africa, which were included in our study. In oral health planning and implementation initiatives, oral cancer burden should be considered presently. However, this study has several limitations. First, our analysis relies on the GBD 2021 study estimates of oral cancer in China, Europe, the US, Southeast Asia and Africa through a time-trend analysis and regional comparison, our findings are derived from this specific subset of regions and may not be fully representative of all global contexts, including other important regions such as South Asia. Second, specific data comparison was only conducted in 2021 rather than each year from 1990 to 2021. The estimation and comparison were not fully and comprehensively from countries and regions. Several other risk factors, including confirmed HPV infection [4] and betel quid without tobacco consumption [26] for oral cancer burden estimates and analysis, were not included in our study. Lastly, our analysis primarily focused on the independent contributions of individual risk factors and did not quantify the known synergistic effects, such as those between smoking and alcohol consumption, which may lead to an underestimation of the true harm associated with combined exposures. In some countries and regions with high incidence and mortality of oral cancer, it is urgent to control exposure to major risk factors along with promoting access to diagnosis and treatment early rather than delaying through conducting public health strategies (e.g., enacting legislation on betel quid control, integrating basic oral examination into primary care, and implementing targeted screening programs). It is crucial that oral health care is given to support early diagnosis and access to treatment of oral cancer in a timely manner [27]. Our results provide evidence for the need of timely oral health care. Identifying oral cancer-specific risk factors, from an assessment of clinical, epidemiological, lifestyle, biological and genetic factors, would contribute to predicting outcomes of cancer care, and facilitate prompt treatment. Moreover, oral cancer is easily detectable in the oral cavity so as to promote its identification early to achieve to better outcomes [28]. Estimating and assessing the risk factors accompanied with differences in clinical epidemiological distribution from this present study would provide important information for designing strategies on implementation of oral health care and prevention.

In conclusion, the burden of oral cancer varied significantly across time and geographic regions. The leading risk factors for mortality and DALYs were directly associated with sex, age, and individual lifestyle factors worldwide. Smoking of older men (≥ 55 years old) in China, alcohol consumption of younger men (from 20 to 54 years old) in Europe and the US, as well as chewing tobacco by individuals especially of women in South-east Asia, may act as critical risk factors for deaths and DALYs due to oral cancer. Our results would contribute to earlier diagnosis, timely access to treatment and to reducing exposure to risk factors to improve outcomes and to reduce the oral cancer burden globally.

## Data Availability

All the data would be supplied from the corresponding authors on reasonable request.
